# Influenza vaccination in pediatric age

**DOI:** 10.1186/1824-7288-41-S2-A30

**Published:** 2015-09-30

**Authors:** Susanna Esposito, Nicola Principi

**Affiliations:** 1 Pediatric Highly Intensive Care Unit, Department of Pathophysiology and Transplantation, Università degli Studi di Milano, Fondazione IRCCS Ca’ Granda Ospedale Maggiore Policlinico, Milan, Italy

## 

Influenza is a very common disease among infants and young children, with a considerable clinical and socioeconomic impact [1]. To reduce the direct and indirect effects of pediatric influenza virus infection, influenza vaccination is recommended worldwide for children considered at risk due to a severe underlying disease. In healthy children influenza vaccination is recommended only in a small number of countries, although guidelines vary regarding the minimum age [2]. In USA universal influenza vaccination in all the age groups is recommended, including all the children until 17 years of age. In UK, influenza vaccination is recommended in the age group 4-17 years, although the program has been activated only recently. Finally, in Canada and other European countries in which health autorithies recommend the vaccine also in the healthy pediatric population, school-age children and adolescents are excluded. However, a large number of European health authorities is still reluctant to include influenza vaccination in their national vaccination programs [2]. The reasons for this reluctance include the fact that the protection offered by the currently available vaccines is considered poor, particularly in younger children. Regarding immunogenicity, younger children are quite similar to the elderly, who, because of the senescence of their immune system, respond poorly to immune stimulation [2]. In both these groups of subjects, both the innate and adaptive immune system are poorly functioning. In particular, B-cells, that are essential for antibody production and immune memory, have limited responses. To increase the immune response of children to inactivated vaccines, a number of measures that have been tested and found to be effective in adults and in the elderly have been studied [3]. The use of adjuvanted vaccines, intradermal (ID) injection, the administration of an increased dose of antigens and the live attenuated influenza vaccine (LAIV) have been evaluated in controlled clinical trials, with good results [3]. Moreover, the possibility of protecting young children through the use of a quadrivalent influenza vaccine (QIV) has been evaluated [3]. None of these measures has been definitively accepted because of the fear of an increased risk of adverse events and because in some instances data regarding immunogenicity and/or clinical efficacy are lacking or are not completely convincing. However, the preliminary data are very interesting in some cases and suggest that some of these measures must be further developed if the problem of the poor protection of young infants has to be solved.
